# Orthorexia Nervosa: A cross-sectional study among athletes competing in endurance sports in Northern Italy

**DOI:** 10.1371/journal.pone.0221399

**Published:** 2019-08-27

**Authors:** Fabrizio Bert, Maria Rosaria Gualano, Gianluca Voglino, Paola Rossello, Jean Paul Perret, Roberta Siliquini

**Affiliations:** 1 Department of Public Health Sciences, University of Turin, Turin, Italy; 2 Degree Course in Dietetics, University of Turin, Turin, Italy; East Stroudsburg University, UNITED STATES

## Abstract

Orthorexia Nervosa (ON) is an eating disorder marked by an excessive control over the quality of the food eaten. Some groups present a higher prevalence of ON and people practicing sports seems to be a population at risk. The aim of this study is to assess the prevalence of ON in endurance athletes and to compare their prevalence with the ones recorded in the sedentary population and in athletes playing other sports. A cross-sectional survey was carried in Piedmont and Valle d’Aosta, among 549 participants in local sports events aged between 18 and 40 years old. The questionnaire assessed socio-demographic characteristics, physical activity, nutrition and diet, the ORTO-15 questionnaire and Eating Habits Questionnaire (EHQ). The sample was stratified according to the minutes of sport practiced in a week and the type of sport played. Crosstab chi-square analyses to determine group differences on categorical variables (e.g. gender), and ANOVAs or t tests to determine group differences on continuous variables were performed. When required, post hoc analyses were performed. Linear and logistic regressions were performed in order to investigate potential predictors of orthorexia. The EHQ mean scores ware significantly higher in people who practice sports >150 minutes/week. EHQ score resulted to be positively correlated with endurance sport practice >150 minutes/week, with a coefficient of 2.407 (I.C.95% [0.27;4.54], p = 0.027). Analyses carried out suggested a correlation between endurance sport practice and ON. Further studies should be performed to identify diagnostic criteria and to compare different questionnaire used to assess them.

## Introduction

For some individuals, healthy eating can be an obsessive concern that becomes the main purpose of life. This condition has been called "Orthorexia Nervosa" (ON), with the prefix "Ortho-" meaning correct, straight or true [1–2]. ON common symptoms are the adherence to extremely restrictive diets, a marked attention in the preparation of food and/or rituals concerning eating. Orthorexic subjects are typically concerned about both the quality and quantity of the foods eaten. They spend a lot of time analysing provenance, manufacturing processes, packaging and the possible impact on their health of the foods they want to buy [3]. These subjects finally become fully dedicated to healthy eating, showing obsessive-compulsive symptoms concerning foods choice and preparation, with serious consequences for physical, psychological and social well-being. Moreover, orthorexics suffer of nutritional deficiencies and weight loss [4]. Bratman emphasizes how ON implies a concern about nutrition that ends up dominating the life of the individual. In orthorexic subjects, less importance is given to personal relationships and careers, together with the progressive appearance of social isolation, anxiety and extreme guilt about the violation of eating rules [1,5]. Symptoms overlay across ON and anorexia nervosa (AN), obsessive-compulsive disorder (OCD), obsessive-compulsive personality disorder (OCPD), somatic symptom disorder, anxiety disorder, and psychotic spectrum disorders [3]. In particular, individuals with ON own similar cognitive traits as individuals with AN and/or OCD. Specifically, perfectionism, impaired external monitoring, cognitive rigidity and traits of anxiety are common characteristics of ON, AN and OCD [3]. Patients affected by ON focus on food quality, showing unrealistic food beliefs, desire to maximize health and flaunt behaviours, while anorexic subjects focus on food quantity, have disturbed body image and drive for thinness [3]. On the other hand, patients affected by OCD present obsessions and compulsions that can go beyond food, realize that behaviours are unreasonable and have ego-dystonic thoughts [3]. Furthermore a study published in 2018 outlined the presence of higher subthreshold autism spectrum symptoms among subjects with psychopathological manifestations of eating disorders [6].

In light of this, some authors consider ON either an emerging eating disorder per se or a possible premorbid symptoms of another eating disorder [7]. Nevertheless, Orthorexia Nervosa is not formally recognized as an eating disorder by the American Psychiatric Association. In fact, scientific community debated on whether orthorexia should be included in the latest edition of Diagnostic and Statistical Manual of Mental Disorders (DSM-5). Finally, no professional consensus was reached and DSM-5 do not include this as a psychiatric diagnosis due to lack of robust empirical data required for proper diagnostic recognition [8].

As ON is not considered a psychiatric diagnosis per se and no standardized diagnosis tool currently exists, its spread is still not clear. Prevalence reported by various studies ranges from 6.9% [9] to 57.6% [10] in samples from the Italian general population and no standardized diagnosis tool currently exists. Literature reports that some groups record a high prevalence of ON, such as professional artists and practitioners of Ashtanga Yoga [11]. Moreover, physicians, medical students and dietitians may be at greater risk of developing ON [5,12]. A relationship between sport or physical activity and higher pathology scores was also found in the evaluation scales for ON [8,13].

In this regard, estimating the prevalence of eating disorders among athletes could be quite complicated. However, the literature suggests that disordered eating behaviours are common among sportsmen [14–16], highlighting the importance of this problem in the athletes community. The prevalence of eating disorder was generally higher among practitioners of sports characterized by weight classes, by strong aesthetic determinants and in sports in which having a reduced body mass is considered advantageous, like several endurance sports [14,15,17]. To the best of our knowledge, only one paper evaluated the ON prevalence in athletes [13]. However, this study, carried out in Italy in 2012, included in the sample only athletes practicing judged sports or fitness activities, while endurance sports (such as running, cycling, rowing, Nordic skiing and triathlon) were not considered. Additionally, there are no clear results on the association between ON and age, gender, education and occupation or BMI [18], suggesting cultural influences in orthorexic behaviours [19].

For these reasons, the present study was conducted with the aim to assess the prevalence of ON in a sample of endurance athletes of two Northern Italy regions (Piedmont and Valle d’Aosta) and to compare this prevalence with the ones recorded in the sedentary population and in the population that practices other sports. Furthermore, the following secondary objectives had been identified:

- Evaluation of the influence of socio-demographic variables such as age, gender, nationality, level of education and employment on the ON prevalence;
- Assessment of the possible association between physical activity and Orthorexia Nervosa, taking into account the type of sport, the time spent in training and the level of practice.

## Materials and methods

### The sample

A cross-sectional survey was carried out in two regions of Northern Italy (Piedmont and Valle d’Aosta) between April and June 2017. STROBE guidelines for observational studies were used for reporting [20]. Only subjects aged between 18 and 40 years old and able to read and understand the questionnaire in Italian language were enrolled in the opportunistic sample, interviewed and therefore included in the study. The sample was recruited among participants (athletes and audience) in local sports events, in particular cyclosportive, running and walking competitions. Participation in the study was voluntary, anonymous and without compensation. The researchers ensured anonymity of participants and the maintenance of ethical principles. In particular, prior to the administration of the survey, the background and the objectives of the study were explained, and subjects were asked to sign an informed consent form. This study was conducted according to the guidelines laid down in the Declaration of Helsinki and all procedures involving human subjects/patients were approved by the Internal Review Board of the Department of Public Health Sciences of the University of Torino, Italy. Written informed consent was obtained from all subjects/patients.

### Measures

The questionnaire was developed after a review of studies on this topic in scientific databases and validated by a pilot study on 20 subjects. The final version was composed by 58 items, grouped in five sections, as follows:

1. Socio-demographic characteristics of the participants interviewed, such as gender, age, nationality, education and occupation [Item 1–8];
2. Physical activity in the last year, such as type of sport performed, number of training sessions and hours of training per week, participation in official competitions [Item 9–15];
3. Nutrition and diet: sources of information, adherence to specific diets, professional point of reference for nutritional follow-up (if any) [Item 16–22];
4. ORTO-15 questionnaire, for the assessment of orthorexia [Item 23–37];
5. Eating Habits Questionnaire (EHQ), for the assessment of orthorexia [Item 38–58].

In particular, to assess nutrition and diet different items were included in the questionnaire. Among them, the presence of dietary restriction was evaluated to define people avoiding specific food for health, religious or ethical reason (E.g.: Vegetarians, gluten free diet, …). On the other hand, being on a diet (in the last 24 months or at the moment of the survey) was assessed to define subjects reducing the food intake to lose or control their weight. These dichotomous variables (possible answers were yes or no) do not overlaps with items from the psychological tests used.

### Psychological tests used

ORTO-15 is the scale used internationally to predict ON [5,21,22]. 15 items, each one with a score from 1 to 4, compose it. A cut-off score of 40 was found to be able to correctly identify the subjects believed to have ON (with a score below 40) [23]. Furthermore, the 21-item Eating Habits Questionnaire (EHQ) assesses the cognitions, behaviours, and feelings regarding healthy eating [2, 24]. For this paper, we used these questionnaires to assess different aspects of this phenomenon. Further information on the questionnaire items, subscale and scoring are given in S1 File.

### Statistical analysis

Statistical analyses were carried out using STATA V.13 (Stata Corp, College Station, Texas, USA, 2013). A descriptive analysis of the sample was conducted and results were expressed in frequencies and percentages for categorical variables or through mean and standard deviation (or median and interquartile range if appropriate) for continuous variables. Statistical significance was assessed through crosstab chi-square analyses to determine group differences on categorical variables (e.g., gender), and ANOVAs or t tests to determine group differences on continuous variables. We decided to perform two different stratifications of the sample. In the first one we stratified according to the minutes of sports carried out during the week (three groups: “No sport”, “Sport<150’” and “Sport≥150’”; the cut-off was chosen according to WHO guidelines for physical activity). Afterwards, we decided to stratify the group of people performing ≥150’ of sport during the week in two subgroups: “Endurance sport<150’” and “Endurance sport≥150’”. Endurance sports were defined as “Sports with predominantly aerobic activity" in the bioenergetics classification of sports proposed by Antonio Dal Monte (1969), revised in 1990 [25]. The evaluation of orthorexia in the above indicated groups was performed by applying the ORTO-15 test, using three possible cut-offs for the identification of potentially orthorexic individuals (40, 35 and 30 points), and also through the EHQ evaluation scale, taken into account both the total score and its three subscales ("Knowledge", "Problems", "Feelings"). The ORTO-15 test results (dichotomized according to the 35 points cut-off) were then used to describe again the sample through a double stratification on the basis of sports activity and of the presence/absence of Orthorexia. These data provided the basis for a multivariate analysis, conducted using linear (ORTO-15 scale used as continuous variable) and logistic (ORTO-15 dichotomized scale) regression models, in order to investigate potential predictors of orthorexia according to the sport and “endurance sport” performed. A similar analysis was then carried out using linear regressions with the EHQ scale results (and its three subscales) as dependent variables. The covariates to be included into the final model were selected with a univariate p value <0.25 as the main criterion. Results are expressed as OR (logistic regression) or regression coefficient (linear regression) with 95% CI, and a two-tailed p value <0.05 was considered significant for all analyses.

## Results

A total of 549 subjects were interviewed, 407 (74.5%) were males and 139 (25.5%) females. In 3 cases gender information was missing. Sample mean age was 26.7± 5.39.

### Sport habits

The minutes spent in a week practicing sports were used to stratify the sample in three groups: 182 subjects (33.2%) declared to be inactive, 47 (8.6%) practice sports for less than 150 minutes/week and 320 (58.3%) were the most active and were classified in the third group “Sport >150’”. There are no statistical differences between the three groups according to mean age, nationality, education or drug use (Table 1). Significant differences were present according to the Body Mass Index (BMI) of the subjects. Subjects who defined themselves as inactive were more likely to be overweight than people who practiced sports less than 150 minutes or more than 150 minutes/week (respectively 13.3%, 4.3% and 10.4%; p = 0.003). Furthermore, the 320 subjects who practice sports more than 150 minutes/week were stratified in two groups according to the minutes spent practicing endurance sports. Ninety-nine subjects declared to practice endurance sports for less than 150 minutes/week, while 221 declared to practice more than 150 minutes/week of endurance sports. No statistical differences were present between the two subgroups according to gender, nationality, education, BMI or drug use. Coherently to the setting where sample was collected, there were more subjects who declared to participate in competition among the group “Endurance sports > 150 minutes/week” than among the other group (71% vs 42.9%; p<0.001).

**Table 1:** Socio-demographic variables of the sample, stratified by minutes of sport activity during the week. Categorical variables are reported as % (Number), while continuous variables are reported as mean ± standard deviation.

	No sport	Sport <150’	Sport ≥150’	p	Endurance <150’	Endurance ≥150’	p
**Gender**							
*Female*	41.01 (57)	12.23 (17)	46.76 (65)	**0.006**^a^	26.15 (17)	73.85 (48)	0.322^a^
*Male*	30.71 (125)	7.37 (30)	61.92 (252)		32.54 (82)	67.46 (170)	
**Mean age**	26.52±5.17	26.38±5.72	26.81±5.47	0.783^b^	25.27±4.74	27.50±5.64	**<0.001**^**c**^
**Nationality**							
*Italian*	33.65 (176)	8.41 (44)	57.93 (303)	0.429^a^	31.35 (95)	68.65 (208)	0.702^a^
*Other country*	21.74 (5)	13.04 (3)	65.22 (15)		26.67 (4)	73.33 (11)	
**Body Mass Index**							
*<18*.*5*	4.42 (8)	8.51 (4)	1.26 (4)	**0.003**^a^	1.01 (1)	1.37 (3)	0.226^a^
*18*.*5–24*.*99*	77.90 (141)	87.23 (41)	87.11 (277)		81.82 (81)	89.50 (196)	
*25–29*.*99*	13.26 (24)	4.26 (2)	10.38 (33)		15.15 (15)	8.22 (18)	
*>30*	4.42 (8)	0.00 (0)	1.26 (4)		2.02 (2)	0.91 (2)	
**Education**							
*Lower degree*	48.90 (89)	42.55 (20)	52.04 (166)	0.438^a^	52.53 (52)	51.82 (114)	0.907^a^
*University degree*	51.10 (93)	57.45 (27)	47.96 (153)		47.47 (47)	48.18 (106)	
**Occupation**							
*Employed*	39.56 (72)	46.81 (22)	54.55 (174)	**0.021**^a^	36.36 (36)	62.73 (138)	**<0.001**^a^
*Student*	53.30 (97)	48.94 (23)	41.69 (133)		60.61 (60)	33.18 (73)	
*Unemployed*	7.14 (13)	4.26 (2)	3.76 (12)		3.03 (3)	4.09 (9)	
**Drug use**							
*Yes*	10.80 (19)	10.87 (5)	5.63 (18)	0.085^a^	7.07 (7)	4.98 (11)	0.452^a^
*No*	89.20 (157)	89.13 (41)	94.38 (302)		92.93 (92)	95.02 (210)	

a: p-value was calculated using crosstab chi-square;

b: p-value was calculated using ANOVA test;

c: p-value was calculated using t-test

### Nutritional habits

Focusing on nutritional habit, there were significant differences between the three groups. Indeed, the 31.6% of the subjects included in the “Sport>150 minutes/week” group declared they had been on a diet in the previous 24 months, while only 25.5% in the group “Sport<150 minutes/week” and 17% in the “No sport” group (p = 0.002) declared the same. In particular, more people who practiced sport for more than 150 minutes/week declared to be on a diet at the moment of the survey, compared with the other two groups (21.7% in the group “Sport ≥150 minutes/week”, 14.9% in “Sport <150 minutes/week” and 6.6% in sedentary people; p<0.001). No significant differences were recorded about nutritional habits between people practicing more than 150 minutes/week of endurance sports and those who practice less (Table 2).

**Table 2:** Nutritional habits of sample, stratified by minutes of sport activity during the week.

	No sport		Sport <150’		Sport ≥150’		p
	% (N)		% (N)		% (N)		
**Dietary restriction**							
*Yes*	10.56	(19)	14.89	(7)	11.29	(36)	0.705**^a^**
*No*	89.44	(161)	85.11	(40)	88.71	(83)	
**Diet in last 24 months**							
*Yes*	17.03	(31)	25.53	(12)	31.56	(101)	**0.002a**
*No*	82.97	(151)	74.47	(35)	68.44	(219)	
**Diet at the moment of survey**							
*Yes*	6.59	(12)	14.89	(7)	21.70	(69)	**<0.001a**
*No*	93.41	(170)	85.11	(40)	78.30	(249)	

a: p-value was calculated using crosstab chi-square. Dietary restriction refers to people avoiding specific food for health, religious or ethical reason (E.g.: Vegetarians, gluten free diet, …). Being on a diet (in the last 24 months or at the moment of the survey) refers to people reducing the food intake to lose or control their weight.

### Evaluation of Orthorexia: The ORTO-15 test

The fourth section of questionnaire [Item 23–37] corresponds to ORTO-15 test. No significant differences were noted fixing a cut-off the score of 40 as suggested in the literature [22]. On the strength of this cut-off, Orthorexia Nervosa was present in 68.8% of “No sport”, in 71.1% of “Sport <150 minutes/week” and in 72.8% of “Sport ≥150 minutes/week” group (Table 3). Fixing the cut-off to 35 points, these percentages were reduced to 19.9%, 24.4% and 21.5% respectively, with no significant differences among the three groups (Table 3). In the “Sport<150 minutes/week” group the mean age of the subjects resulting orthorexic with a cut-off of 35 was significantly lower (23.2±3 vs 27.7±6, p = 0.002). Moreover, in this group there were evidence that the ON symptoms were uncommon in workers (9.1%) but frequent in students (42.8% orthorexic p = 0.026). ORTO-15 final scores were also analysed as continuous variable, without cut-off, in order to find potential predictors of Orthorexia Nervosa: the adhesion to a dietary treatment plan in the two previous years was negatively correlated with test score (coeff = -1.515, I.C.95% [-2.26–0.77], p<0.001), while no statistically significant correlation was found between ORTO-15 score and the other sociodemographic and behavioural variables.

**Table 3:** Sample mean ORTO-15 and Eating Habit Questionnaire scores, based on sport activity during the week.

	No sport	Sport <150’	Sport ≥150’	p
**ORTO-15**				
Mean ±SD	37.59±3.63	37.38±4.07	37.26±3.75	0.654^a^
**ORTO-15**	**% (N)**	**% (N)**	**% (N)**	
Cut-off 40 points	68.75 (121)	71.11 (32)	72.76 (227)	0.643^b^
Cut-off 35 points	19.89 (35)	24.44 (11)	21.47 (67)	0.787^b^
Cut-off 30 points	1.74 (3)	4.44 (2)	1.65 (5)	0.435^b^
**EHQ total score**				
Mean ±SD	18.78±8.39	23.46±7.18	23.81±9.14	**<0.001**^**a**^
**EHQ knowledge**				
Mean ±SD	6.72±2.83	7.96±2.76	8.23±2.91	**<0.001**^**a**^
**EHQ Problems**				
Mean ±SD	5.56±5.10	8.11±4.66	8.22±5.48	**<0.001**^**a**^
**EHQ Feelings**				
Mean ±SD	6.44±2.38	7.25±2.32	7.33±2.31	**<0.001**^**a**^

a: p-value was calculated using ANOVA test:

b: p-value was calculated using crosstab chi-square. SD: Standard Deviation; EHQ: Eating Habit Questionnaire.

### Evaluation of Orthorexia: The Eating Habit Questionnaire

The fifth section of questionnaire [Item 38–58] corresponds to the EHQ test. No scoring system or cut-off were found in literature. In this analysis, “Very true” answers were scored as 3, “Mainly true” answers were scored as 2, “Slightly true” answers were scored as 1 and “False/Not at all” answers were scored as 0. Final scores were analysed as continuous variable without cut-off and the sub-scales assessment was performed according to the three sub-scales “Knowledge”, “Problems” and “Feelings”. We found significant differences between the three groups analysed (p<0.001). In particular, the mean scores were significantly higher in people performing sports >150 minutes/week (23.8±9.1) and “Sport <150 minutes/week” (23.7±7.2) than in “No sport” group (18.8±8.4). Similar results were highlighted assessing the sub-scales (Table 3). Furthermore, a multiple linear regression model was performed in order to identify potential predictors of EHQ score (and consequently of orthorexic behaviour).

Statistically significant correlations was found relating EHQ score, analysed as continuous variable, and the three groups “No sport” “Sport <150 minutes/week” “Sport ≥150 minutes/week”. Male gender appeared negatively correlated with EHQ score (coeff = -1.813, I.C.95% [-3.49;-0.14], p = 0.034), while there was a positive correlation for the following variables: to be on a diet in the previous two years (coeff = 6.200, I.C.95% [4.56;7.84], p<0.001), the adoption of dietary restrictions in the previous two years (coeff = 4.914, I.C.95% [2.65;7.17], p<0.001), the regular physical activity lower than 150 minutes/week (coeff = 3.614, I.C.95% [0.98;6.25], p = 0.007) and higher than 150 minutes/week (coeff = 4.054, I.C.95% [2.49;5.61], p<0.001). No statistically significant correlation was found relating EHQ score and other sociodemographic and behavioural variables (Table 4). Moreover, considering the two groups “Endurance sport<150 minutes/week” and “Endurance sport>150 minutes/week”, EHQ score resulted positively correlated with the second group with a coefficient of 2.407 (I.C.95% [0.27;4.54], p = 0.027)

**Table 4:** Potential predictors of Orthorexia Nervosa evaluated by Eating Habit Questionnaire (continue variable), based on endurance activity minutes during the week.

		Coefficient	95% CI	p-value^a^
**Gender**	*Female*	1		
	*Male*	-1.813	-3.49; 0.14	**0.034**
**Mean age**		-0.053	-0.23; 0.13	0.563
**Body Mass Index**	*18*.*5–24*.*99*	1		
	*25–29*.*99*	-0.499	-2.80; 1.80	0.670
	*>30*	-3.418	-8.11; 1.27	0.153
	*<18*.*5*	-0.585	-4.85; 3.67	0.787
**Education**	*Lower degree*	1		
	*University degree*	0.967	-0.49; 2.43	0.194
**Occupation**	*Employed*	1		
	*Student*	-1.072	-3.02; 0.87	0.279
	*Unemployed*	-1.186	-4.46; 2.09	0.477
**Diet in last 24 months**	*No*	1		
	*Yes*	6.200	4.56; 7.84	**<0.001**
**Endurance time (minutes)**	*0*	1		
	*<150*	3.614	0.98; 6.25	**0.007**
	*>150*	4.054	2.49; 5.61	**<0.001**
**Dietary restriction**	*No*	1		
	*Yes*	4.914	2.65; 7.17	**<0.001**

**a**: p-value was calculated using multiple linear regression model. Dietary restriction refers to people avoiding specific food for health, religious or ethical reason (E.g.: Vegetarians, gluten free diet, …). Being on a diet (in the last 24 months or at the moment of the survey) refers to people reducing the food intake to lose or control their weight.

## Discussion

There are many international studies trying to assess the prevalence of Orthorexia Nervosa in different sub-populations [5,7–10,12,25] and to describe its different aspects [1–4]. During time many assessment tools have been developed to identify subjects affected by ON. The first was a 10-item scale developed in 2000 by Bratman and Knight [26]. Successively ORTO-15 has been developed and it is nowadays the most commonly used scale to predict ON and it has been translated in different languages [5,8,21,27–32]. A cut-off score of 40 was fixed because it had been considered predictive of ON [9,23]. Further studies have criticized ORTO-15 and showed that a 35 cut-off, can maximize either sensitivity and specificity [10,33]. Although most of the studies used ORTO-15 scale, its validity and reliability varied significantly during time. The prevalence recorded ranged from 4% [23] to 86% [11]. Additionally, according to a paper published in 2017, the mean Cronbach’s alpha, is 0.55, below the standard 0.7 for research studies and 0.9 for diagnostic uses. [34]. In order to assess ON, the urge for a reliable tool becomes increasingly more compelling. The 21-item Eating Habits Questionnaire (EHQ) has been designed to assess the cognitions, behaviours, and feelings related to an extreme focus on healthy eating [2,24]. Based on the first set of papers, the results seem encouraging: Cronbach's alpha was 0.90 for the total composite score for the EHQ, 0.87 for the EHQ-Behaviours subscale, 0.79 for the EHQ-Problems subscale, and 0.73 for the EHQ-Feelings subscale [34]. On the other hand, no clear cut-off are given for the EHQ scale. Considering strength and limitation of each tools, we used these two different scales proposed by other authors to assess this phenomenon and its potential predictors, comparing three different groups according to the minutes/week spent practicing sports. The first evaluation tool used, the ORTO-15 test, showed no significant differences among groups according to gender, education or BMI, while having nutritional restriction in the previous two years seemed to be a predictor of developing ON. These results agree with the results of two review published in 2008 and 2017 [24,35] but are not comparable to the results from other studies [10]. Furthermore, no significant differences were found between inactive people and athletes. There is no agreement between this results and the ones recorded in international studies [13], but probably a different classification of “athlete” can explain these differences. Many studies have criticized the ORTO-15 questionnaire and its cut-off [10,33]. In particular recent studies have shown that a 35 cut-off, instead of 40, can maximize both sensitivity and specificity [33]. In this case, fixing the cut-off at 40, we registered a prevalence of ON around 70%. Fixing the cut-off at 35, the prevalence decreased to 22%, similarly to the results presented in other international studies [10,33].

On the contrary, the Eating Habit Questionnaire (EHQ) has shown significant differences according to gender. In particular, young women seem more easily affected by ON than young men. More noticeable differences were shown between inactive people and athletes. Higher scores were recorded, indeed, in people practicing sports more than 150 minutes/week. At the same time, people practicing endurance sports more than 150 minutes/week have scores suggesting a higher probability to develop ON than people practicing other sports. This results, confirmed in the questionnaire sub-scales, are in accordance with previous papers. In fact, prior studies outlined higher prevalence of eating disorders among athletes compared to control groups [14–17]. In particular, higher prevalence was recorded among athletes participating in sports where low body weight or leanness confers a competitive advantage. For these athletes the weight loss leads to a better performance and this can push them to continue dieting and slip into an eating disorder [15,16]. Additionally, assessing 411 university students, ON symptomatology was found to be associated with aerobic exercise levels, exercise addiction and exercise motivation [24].

Furthermore, the existing literature points out the need for further research into the impact of BMI on ON, In fact, mixed findings can be recorded when reading the previous results. Some papers showed a positive correlation between ON symptomatology and BMI [21,34]. On the contrary, others studies found no significant relationship [8–10]. The present study found no association between the Body Mass Index and ON symptomatology, but coherently to previous paper a negative correlation between BMI and ON has not found, suggesting that ON is distinct from AN, which is marked by an excessively low BMI [34].

Similarly, the literature is not unanimous about gender differences in ON symptomatology. In fact some studies showed a higher prevalence in women [30], others in men [9] and others show no significant differences in ON symptomatology [10,11].

Interestingly, there are some differences according to socio-economical and health variables due to the amount of sport practiced. Surprisingly, more subjects are overweight in the group practicing sports more than 150 minutes/week than in the one practicing sports less than 150 minutes/week. This can be explained considering that BMI is related to weight but does not consider the proportion of Fat Mass and Fat Free Mass. In addition, in our sample, women are generally more inactive than men and employed are more likely to practice sports than students/unemployed. This could be explained considering the possibility to manage free time.

It has to be stated that this study has some limitation. First of all, convenience sample can represent a weakling point. For example, the opportunistic sampling strategy could explain most of the subjects interviewed are male. These could be a limitation, but gender in our sample is not effecting the results of the ORTO-15 nor of the EHQ scales, coherently to what has been registered in previous studies [5,9,23,30]. Additionally, the participants in the study are aged between 18 and 40. This decision was taken to reduce possible age differences among the sport active group and the one who does not practice sports, but reduce the representativeness to this specific age group. On the other hand, Orthorexia Nervosa is an emerging issue and no validated diagnostic criteria are defined [4, 36]. Prevalence in general population varies in different studies [9–12] and no clear risk factor are identified. For these reason to identify proper inclusion/exclusion criteria was demanding and we decided to be as inclusive as possible, considering the need to assess ON prevalence in general population [3]. It has to be stated that the evaluation scales have some weakness, in particular there is no clear statement about the cut off to use in the ORTO-15 scale [10,33] and EHQ has been used in few studies and has not been validated. For these reason these scales can highlight specific condition concerning eating habits, but cannot be considered diagnostic tools.

Further studies are required to assess ON in order to better define ON and develop proper diagnostic tool. In fact, it seems clear that among people affected by ON a positive attention to an healthy and balanced diet twists into endangering food restrictions and eating disorders [1,5,36]

## Conclusions

In conclusion, from these analyses emerge how physical activity and, in particular, endurance sports seems to be connected with Orthorexia Nervosa. In addition, having a nutritional plan or nutritional restriction seems to be connected with ON. Further studies need to be performed either to identify criteria to define ON either to compare different questionnaires used to assess it. ON is indeed an emerging issue that can have serious consequences on athletes’ health and that involves in its solution health professionals with different competences, such as nutritionists, psychiatrists, sociologists and public health professionals.

## Supporting information

S1 File Scoring grid for ORTO-15 questionnaire and Eating Habit Questionnaire (EHQ) items and subscale. K: Knowledge; P: Problem; F: Feeling.
(DOCX)
