# Supplementary material for: Orthorexia Nervosa: A cross-sectional study among athletes competing in endurance sports in Northern Italy
Source: PLoS One. 2019 Aug 27;14(8):e0221399. doi: 10.1371/journal.pone.0221399 (PMC6711511; doi:10.1371/journal.pone.0221399)
Supplement: S1 File — K: Knowledge; P: Problem; F: Feeling. (DOCX) [file pone.0221399.s001.docx]

**S1 Table: Scoring grid for ORTO-15 questionnaire**

| **Items** | **Scoring Grid** | | | |
| --- | --- | --- | --- | --- |
|  | **Always** | **Often** | **Sometimes** | **Never** |
| 1. *When eating, do you pay attention to the calories of the food?* | 2 | 4 | 3 | 1 |
| 1. *When you go in a food shop do you feel confused?* | 4 | 3 | 2 | 1 |
| 1. *In the last 3 months, did the thought of food worry you?* | 1 | 2 | 3 | 4 |
| 1. *Are your eating choices conditioned by your worry about your health status?* | 1 | 2 | 3 | 4 |
| 1. *Is the taste of food more important than the quality when you evaluate food?* | 4 | 3 | 2 | 1 |
| 1. *Are you willing to spend more money to have healthier food?* | 1 | 2 | 3 | 4 |
| 1. *Does the thought about food worry you for more than three hours a day?* | 1 | 2 | 3 | 4 |
| 1. *Do you allow yourself any eating transgression?* | 4 | 3 | 2 | 1 |
| 1. *Do you think your mood affects your eating behaviour?* | 4 | 3 | 2 | 1 |
| 1. *Do you think that the conviction to eat only healthy food increases self-esteem?* | 1 | 2 | 3 | 4 |
| 1. *Do you think that eating healthy food changes your life-style (frequency of eating out, friends, …)?* | 1 | 2 | 3 | 4 |
| 1. *Do you think that consuming healthy food may improve your appearance?* | 1 | 2 | 3 | 4 |
| 1. *Do you feel guilty when transgressing?* | 2 | 4 | 3 | 1 |
| 1. *Do you think that on the market there is also unhealthy food?* | 1 | 2 | 3 | 4 |
| 1. *At present, are you alone when having meals?* | 1 | 2 | 3 | 4 |

**S2 Table. Eating Habit Questionnaire (EHQ) items and subscale.**

| **Items** | **Subscale** | **Scoring Grid** | | | |
| --- | --- | --- | --- | --- | --- |
|  |  | **False/ Not at all** | **Slightly True** | **Mainly True** | **Very true** |
| *1. I am more informed than others about healthy eating* | K | 0 | 1 | 2 | 3 |
| *2. I turn down social offers that involve eating unhealthy food.* | P | 0 | 1 | 2 | 3 |
| *3. The way my food is prepared is important in my diet.* | K | 0 | 1 | 2 | 3 |
| *4. I follow a diet with many rules.* | K | 0 | 1 | 2 | 3 |
| *5. My eating habits are superior to others.* | K | 0 | 1 | 2 | 3 |
| *6. I am distracted by thoughts of eating healthily.* | P | 0 | 1 | 2 | 3 |
| *7. I only eat what my diet allows.* | K | 0 | 1 | 2 | 3 |
| *8. My healthy eating is a significant source of stress in my relationships.* | P | 0 | 1 | 2 | 3 |
| *9. I have made efforts to eat more healthily over time.* | F | 0 | 1 | 2 | 3 |
| *10. My diet affects the type of employment I would take.* | P | 0 | 1 | 2 | 3 |
| *11. My diet is better than other people’s diets.* | K | 0 | 1 | 2 | 3 |
| *12. I feel in control when I eat healthily.* | F | 0 | 1 | 2 | 3 |
| *13. In the past year, friends or family members have told me that I’m overly concerned with eating healthily.* | P | 0 | 1 | 2 | 3 |
| *14. I have difficulty finding restaurants that serve the foods I eat.* | P | 0 | 1 | 2 | 3 |
| *15. Eating the way I do gives me a sense of satisfaction.* | F | 0 | 1 | 2 | 3 |
| *16. Few foods are healthy for me to eat.* | P | 0 | 1 | 2 | 3 |
| *17. I go out less since I began eating healthily.* | P | 0 | 1 | 2 | 3 |
| *18. I spend more than three hours a day thinking about healthy food.* | P | 0 | 1 | 2 | 3 |
| *19. I feel great when I eat healthily.* | F | 0 | 1 | 2 | 3 |
| *20. I follow a health-food diet rigidly.* | K | 0 | 1 | 2 | 3 |
| *21. I prepare food in the most healthful way.* | K | 0 | 1 | 2 | 3 |

*K: Knowledge; P: Problem; F: Feeling*.
